# Complex Case Management Workshop for Trainees &Trainers - a Quality Improvement Project -to Enhance the Understanding of the Case Management and Complexity in People With Intellectual Disabilities for Senior Trainees and Early Career Consultants

**DOI:** 10.1192/bjo.2022.302

**Published:** 2022-06-20

**Authors:** Syeda Hasan, Mark Spurrell

**Affiliations:** 1 Pennine Care NHS Foundation Trust, Manchester, United Kingdom; 2 Mersey Care NHS Foundation Trust, Manchester, United Kingdom

## Abstract

**Aims:**

Whilst there is always more to learn within any speciality, the particular zone of development for senior trainees and early career consultants is the management of the complex case. The factors underpinning complexity are varied, and can range from aspects of the service user such as complexity and severity of disorder and/or multi-morbidity, to aspects of their local social networks, to complexities arising from the service or care environment. The aim of this workshop was to offer a safe space for senior trainees and early career consultants to work through some principles that might be helpful, particularly where things seem to be getting into a pickle.

**Methods:**

- A 60-minute slot was proposed, as part of the Northwest Learning disability academic teaching forum.
- The structure of the workshop borrowed the informality and spirit of support from Balint groups.
- Attendees were prepared to talk about one of their cases anonymously, and why they might be getting into a pickle.
- Soundings were taken and a particular case chosen to workshop in more detail, as an exemplar for others.
- Each session focused on one or two themes from the wider set of themes suggested by the CCaRM framework (Spurrell, Potts & Shaw, 2019) currently being used by Greater Manchester and Lancashire SST to expedite their work.
- Each session concluded with an evaluation discussion of workshop usefulness.

**Results:**

- There were 6 virtual workshops in first PDSA cycle (June 2021-Nov 2021)
- Each workshop lasted approximately 100–120 minutes, with 8–12 people attended in each session.
- The intention for the workshop was to include senior trainees and early career consultants who are within the NWLD forum however junior trainees, senior consultants and other MDT professional also became part of the sessions.
- Each session concluded with an evaluation discussion of workshop usefulness
- Qualitative feedback has been collated and analysed from the evaluation discussion.
- The information derived from the qualitative analysis indicates that there is a substantial need for regular complex case discussions for the purposes of enhanced care delivery and improved patient outcomes

**Conclusion:**

This approach helped in enhancing the understanding of the case management for people with intellectual disabilities among forum members

We consider that the online workshops is a success and are planning further-PDSA 2 in order to create competent and well-rounded ID psychiatrist in the future in Northwest region.

